# Exploring the relationship between lay theories of gender and attitudes to abortion in the context of a national referendum on abortion policy

**DOI:** 10.1371/journal.pone.0218333

**Published:** 2019-06-13

**Authors:** Cliodhna O’Connor, Paul Maher, Irini Kadianaki

**Affiliations:** 1 School of Psychology, University College Dublin, Dublin, Ireland; 2 Department of Psychology, University of Limerick, Limerick, Ireland; 3 Department of Psychology, University of Cyprus, Nicosia, Cyprus; Middlesex University, UNITED KINGDOM

## Abstract

The relationship between lay theories of gender and attitudes to abortion policy has received minimal empirical attention. An ongoing theoretical debate in the psychological essentialism literature queries whether biological attributions causally influence social attitudes or primarily function to justify existing attitudinal commitments. The current research used the context of a national referendum on abortion in Ireland to investigate whether endorsement of certain gender theories is contingent on their rhetorical construction as supporting particular attitudes to abortion. Two experimental studies were conducted online in the three weeks preceding the Irish abortion referendum. The studies tested whether participants would adapt their causal gender beliefs after reading that biological (Study 1; *N* = 348) or social (Study 2; *N* = 241) accounts of gender supported or conflicted with their intended vote in the referendum. Both studies showed the opposite effect: causal gender theories presented as conflicting with participants’ voting intentions subsequently showed elevated support, relative to theories that purportedly aligned with participants’ voting intentions. While results confirm that lay theories of gender are mutable, the direction of effects does not support the proposition that gender theories are selectively endorsed to support existing socio-political attitudes to abortion. Potential mechanisms for the results observed are discussed.

## Introduction

Abortion remains one of the most contentious political issues in societies across the world. In political discourse, abortion is often characterised as an intrinsically feminist issue. However, relatively little research clarifies how the general public’s attitudes to abortion are related to specific understandings of gender. Social psychological research has shown that numerous socio-political attitudes and behaviour are predicted by lay theories of gender–‘common sense’ explanatory frameworks that capture people’s causal attributions about the origin of gender differences. While people who orient towards a biological theory of gender view sex categories as strictly binary, naturally-given and immutable, those with a social theory view gender as a more fluid, socially constructed dimension. This paper explores the ways abortion attitudes intersect with causal beliefs about gender categories, within the unique social context of a national referendum held to legalise abortion in the Republic of Ireland.

### Lay theories of gender

Psychological essentialism refers to the tendency to define social categories in terms of an underlying causal ‘essence’ that makes category membership seem immutable, discrete and internally homogeneous [1,2]. Across a range of dimensions, including gender, race, mental illness and obesity, essentialist constructions of social groups are linked with more negative attitudes towards those groups [3]. While essentialism has been defined in many different ways [2], one consistently important component is the extent to which group differences are attributed to innate biological factors versus social experience [1]. The biological dimension of essentialism is particularly significant in the domain of gender, where causal attributions have important implications for social attitudes and behaviour. Research links biological explanations of gender with greater perception of gender differentiation [4,5], endorsement of gender stereotypes [6,7], sexist attitudes [8], acceptance of inequalities [9,10], reduced support for women’s rights [11], and disfavouring of female political candidates [10]. Additionally, biological explanations of sex roles can operate as ‘self-fulfilling prophecies’ by establishing gendered norms that people gradually internalise. Among women, encountering biological theories of gender prompts greater identification with negative feminine stereotypes [12] and impaired mathematics performance [13–15]. Meanwhile, research with male participants has found that men who mistakenly believe they have ingested testosterone behave more selfishly [16], thereby reproducing the stereotypical association of masculinity with more self-interested behaviour [17,18].

Most research linking biological gender theories to conservative social attitudes is correlational, which makes the direction of causality between attitudes and causal attributions ambiguous. Conventionally, essentialist beliefs are conceptualised as causal antecedents of attitudes regarding gender issues [19]. However, there is also empirical and theoretical precedent for considering essentialist attributions as post-hoc rationalisations of pre-existing attitudes or ideologies. Some have proposed that essentialist ideas operate as a form of system justification, by making unequal social arrangements seem just and inevitable [9,20]. More prejudiced people spontaneously generate more causal attributions about stigmatised groups’ attributes, suggesting that in a socio-political context that discourages overtly derogatory aspersions about minority groups, biological attributions may function to justify prejudice [19]. Experimental studies indicate men with sexist beliefs are more likely to endorse gender essentialism when they are led to believe the gender status quo is changing [9]. Similarly, when heterosexual males’ privileged social status is experimentally threatened, they seek to preserve their group’s distinctiveness by increasing belief in biological causation of sexual orientation [21]. Men and women show higher support for scientific research showing biologically-determined sex differences when the findings purportedly prove the superiority of their own gender [22]. These studies suggest that causal attributions can be motivated by particular attitudinal and identity commitments, rather than vice versa.

A key advantage biological essentialism offers for motivated social cognition is its flexibility. While carrying the semblance of objective factual information, essentialist statements can be rhetorically leveraged to justify diverse, even antithetical positions [9,23]. For example, essentialist representations of cultural groups can be recruited to support both exclusive and inclusive models of citizenship [24]. Sexual orientation beliefs also demonstrate the multifarious functionality of biological essentialism: although belief in the innateness of sexual orientation typically correlates with more positive attitudes to gay rights [25], the notion that homosexuals represent a discrete biological ‘kind’ can also sanction their othering and marginalisation [26–28]. Research exploring the biological attributions that occurred in public discourse preceding a 2015 referendum on marriage equality in Ireland confirmed that both sides of the debate used ‘appeals to nature’ to justify their position: opponents of marriage equality emphasised the biological basis of parenthood and gender; supporters emphasised the biological basis of homosexuality; and both sides coincided in valorising marriage itself as a naturally desirable state [29]. The influence of exposure to such arguments on the public’s attitudes or baseline levels of essentialist beliefs remains unclear.

### Abortion attitudes and gender beliefs

Although existing research confirms that biologically essentialist attributions are recruited in public discourse to serve a range of rhetorical functions [6,24,29], and have generally (although not exclusively) negative effects for intergroup relations [26], limited work has charted the specific, real-world socio-political issues with which they interact. For instance, little published research to date has explored the relationship between lay theories of gender and attitudes to abortion policy. Public opinion research shows abortion attitudes are influenced by a range of variables, including age [30,31], religion and religiosity [32–38,38,39], education [40,41], political affiliation [35,42–46] and personal values [32,35,36,38]. Most of this evidence comes from sociological or political science studies of socio-demographic predictors, with less attention to the social psychological dynamics of public attitudes to abortion [47–49]. Moreover, the vast majority of research on abortion attitudes emanates from the US. Given that the meanings surrounding abortion are intrinsically cultural [50–53], greater diversity of research contexts is warranted. The social psychological predictors of abortion attitudes in the Republic of Ireland, the setting for the current research, have not previously been studied.

The role played by gender in abortion attitudes is complex. Reproductive rights have long been a key cause of feminist movements worldwide [54,55] and research from the 2000s onwards tends to show women more supportive of abortion rights [30,36,39,56]. However, gender itself is not a strong or consistent predictor of abortion attitudes [57]; more important are the beliefs that an individual holds about gender roles and relations. Endorsement of the value of gender equality is consistently related to more liberal abortion attitudes [38,54,56], and international research has identified correlations between anti-abortion attitudes and traditional beliefs about gender roles [35,36,38,39,46,56,58,59]. However, Jelen and Wilcox’ [41] review suggests these statistical relationships often disappear once the effects of religious and political affiliation are controlled. Moreover, the strength of the relationship between gender role beliefs and abortion attitudes vacillates across cultural contexts, with the relationship particularly weak in countries with restrictive abortion laws [60]; this suggests there is no inevitable correspondence between the two attitudinal domains [39,60]. In most Western countries, gender role beliefs changed profoundly in the latter half of the twenty-first century, as did normative sexual morality regarding issues like extramarital sex and same-sex marriage [41]. Abortion attitudes have not undergone the extent of liberalisation that would be expected if they were causally linked with gender role beliefs [41,61]. Indeed, there is some evidence that aggregate attitudes to abortion in the US shifted in a more conservative direction in the late 20^th^ century, even as attitudes towards gendered divisions of labour grew more egalitarian [41].

Notably, the strength of the relationship between gender role beliefs and abortion attitudes has also declined over time [60]. This may be related to increasing consensus on gender role beliefs, or at least on the beliefs that are acceptable to express, which have reduced meaningful variation in standardised measures. A more valid construct for contemporary contexts is ambivalent sexism, which acknowledges that contemporary gender inequalities are perpetuated by a combination of old-fashioned ‘hostile’ sexism and modern ‘benevolent’ sexism. While hostile sexism predicts attitudes to cases of sexual harassment, abuse and assault [62–65], benevolent sexism predicts victim-blaming, paternalism and harsh judgement of female sexuality [62,66–68]. Studies confirm that ambivalent sexism predicts opposition to abortion in the general population, with benevolent sexism a stronger influence than hostile sexism [69,70]. Research further suggests the component of benevolent sexism that critically mediates the link to abortion attitudes is the idealisation of motherhood [71]. With regard to the present study, it is notable that this component of benevolent sexism articulates an essentialist view of gender differences, incorporating the belief that women’s biological role in human reproduction endows them with distinct emotional and behavioural attributes. Further indications that biological beliefs may relate to abortion attitudes come from qualitative analyses of pro-life discourse, which suggest that anti-abortion attitudes are often informed by a belief in sexual ‘complementarity’ that constructs men and women as distinct natural ‘types’ and conflates female identity with a natural maternal instinct [55,72]. Interestingly, gender essentialism can also surface in pro-choice discourse, particularly in the argument that (cisgender) men, due to their biological incapability of experiencing pregnancy, are unqualified to make judgements on a woman’s reproductive choices [73]. Abortion thus seems to be one socio-political issue where the flexible rhetorical properties of biological gender essentialism are exploited. This offers an opportunity to advance understanding of how causal gender attributions are woven into socio-political discourse and the consequences for public attitudes and beliefs. To date, no published quantitative research has explored how lay theories of gender relate to abortion attitudes.

### Research context: The Irish abortion referendum

Understanding the social psychological dynamics of public attitudes to abortion is particularly important in the many regions of the world where abortion legislation remains a matter of active contention. One such jurisdiction is the Republic of Ireland. Until 2018, Ireland maintained one of the most restrictive abortion regimes in Europe, permitting termination only when necessary to prevent serious risk to the mother’s life. The legal framework governing this was the Eighth Amendment of the Irish Constitution, which committed the State to defend “the right to life of the unborn” and was sanctioned by 66.9% of the voting public in a 1983 referendum. The ensuing decades saw abortion remain a fiercely contested issue, with the hazards of the abortion ban highlighted by a series of medical tragedies, high-profile civil and Supreme Court cases, and condemnation from the international courts and human rights community [74]. Following the 2013 death of Savita Halappanavar, who died of sepsis after being refused a termination in an Irish hospital, serious political momentum around changing Ireland’s abortion legislation began to coalesce. As any change to the Irish Constitution requires majority approval by the population, a referendum to repeal the Eighth Amendment was called for 25^th^ May 2018. The repeal proposition was supported by all major political parties and most civil society organisations and, after a heated public debate, passed with 66.4% of the vote (voter turn-out was 64.5%, the 3^rd^ highest ever in an Irish referendum). Legislation to permit abortion up to 12 gestational weeks, and in select circumstances thereafter, was approved in 2018.

Social psychological research on public attitudes is most valid when the attitude domain is topical in a particular socio-political context and has immediate behavioural implications with high material stakes. As a case where citizens were highly engaged with a pressing public issue, and obliged to make a binary voting decision that would dictate national legislation and practice, the Irish referendum offered a singular empirical opportunity to explore the social psychological dynamics of abortion attitudes. The current research used this unique historical context to investigate the nature and directionality of the relationship between causal theories of gender and attitudes to abortion policy.

The aim of the research was to explore the motivated dimension of causal gender attributions by experimentally investigating whether presenting particular gender theories as consistent vs. inconsistent with an important socio-political attitude (voting intention in the Irish abortion referendum) would shift people’s endorsement of causal gender attributions. Demonstration of the mutability of gender beliefs would contribute to the theoretical debate regarding whether attributions causally influence social attitudes, or primarily function to justify existing attitudinal commitments [9,19,75]. The research consisted of two studies (total *N =* 589) conducted in the three weeks preceding the referendum (4^th^-25^th^ May 2018). Participation was restricted to those who were eligible to vote in the referendum (i.e., Irish citizens over 18 years old). Both studies were conducted in line with national, professional and institutional research ethics policies and participants gave informed consent to participate.

## Study 1

Study 1 sought to clarify the relationship between abortion attitudes and biological gender beliefs through an experimental manipulation that informed participants that a biological theory of gender was either consistent or inconsistent with their stated voting intention in the abortion referendum. If endorsement of the biological theory was lower following its positioning as inconsistent with one’s existing attitude to abortion, this would provide evidence that lay gender theories are mutable to attitude justification motives.

### Method

#### Design

An online experiment was run on Qualtrics. After consenting to participate by ticking a box, participants were asked to indicate their voting intention in the upcoming referendum and their level of certainty (on a 4-point scale) they would vote this way. The survey programme then randomly assigned participants to read one of two 201-word passages (S1 Appendix). Both passages shared a common first section, which strongly asserted the biological origins of gender. The passages then diverged in building an argument that the biological aetiology of gender supported either a No (pro-life) or Yes (pro-choice) vote. The passages were developed following an inspection of media content to identify common ways biological construals of sex/gender were absorbed into arguments for and against abortion. Previous scholarly analyses of Irish abortion discourse were also consulted [73,74,76].

After reading the passage, participants were asked to evaluate the strength of its argument on a 5-point scale. The next page included an attention/memory check that asked participants whether the passage they had read favoured a No or Yes vote. This was followed by the Lay Gender Beliefs scale [12], an 11-item scale comprising two subdimensions that measure endorsement of a Biological Theory (Cronbach’s α = .84) and Social Theory (α = .80) of gender. The order of items on this scale was randomised. The questionnaire concluded with a battery of socio-demographic questions. Participants were then fully debriefed regarding the study aims and design.

#### Participants

Participants were recruited by circulating adverts with links to the study through social media and popular Irish web forums. As the study involved a test of motivated reasoning, recruitment targeted online communities and discussion groups likely to be frequented by those invested in the referendum debate (e.g. referendum-themed groups on Facebook, referendum-themed threads on forums including reddit.com/r/ireland/, politics.ie, politicalirish.com, irishcatholics.proboards.com, rollercoaster.ie). As younger people, who tend to be more pro-choice, are more likely to be active on social media, specific effort was made to identify online communities that favoured a No vote (e.g. religiously affiliated groups and anti-abortion forum discussion threads). The research was introduced as a study of the factors that influence voting intentions and attitudes to abortion policy.

A total of 348 people opted into the study. Participants were aged between 18–68, with a mean of 30.41 years (*SD =* 10.69). Of those who stated their gender, 65% (*n* = 178) were female. Over half (58.2%; *n* = 163) were single, 40% (*n* = 112) were married or cohabiting, and 1.8% divorced/separated/widowed (*n* = 5). Approximately two-thirds (67.6%, *n* = 190) were not parents. 60.1% (*n* = 169) had university-level education. In an open question asking participants to define their religion, 61.7% (*n* = 158) stated they had none or identified as atheist, agnostic or ‘non-practicing’; one-third (34%, *n* = 87) identified as Christian, with the majority being Catholic. Levels of religiosity were low across the sample, with a mean of 1.66 (*SD =* 1.07) on a 5-point scale (1 = ‘not at all important’, 5 = ‘extremely important’). Mean political orientation on a 5-point scale (1 = ‘extremely liberal’, 5 = ‘extremely conservative’) was 2.17 (S*D =* .99).

#### Analysis

Data were downloaded from Qualtrics and entered into SPSS for cleaning and analysis. Cases with no valid data were excluded. All other partial responses were included, with missing data within individual analyses excluded listwise (as a result, the sample sizes for the tests reported below differ depending on data availability for relevant variables). Correlations and *t*-tests were used to explore the relationship between sociodemographic variables and lay gender theories. An ANCOVA tested the hypothesis that people’s endorsement of Biological Theory would be stronger following the presentation of a biologically essentialist construction of gender as supporting their voting intention (i.e. significant interaction between participant’s voting intention and direction of the argument they read). A further ANCOVA explored the effect of the same experimental manipulation on the Social Theory subscale. All tests were two-tailed.

### Results

#### Voting intentions

At the time of data collection, 76.7% (*n =* 267) intended to vote Yes, 18.7% (*n =* 65) No and 4.6% (*n =* 16) were undecided. When asked to rate their certainty they would vote this way, 90.3% (*n =* 241) of Yes voters and 73.4% (*n =* 47) of No voters self-classified as ‘absolutely certain’.

Qualtrics randomly assigned 148 people (46.5%) to read the passage favouring a No (anti-abortion) vote and 170 (53.5%) a Yes vote. A two-way ANOVA (excluding participants who failed the attention/memory check) was conducted to assess whether the two variants of the passage were equivalent in perceived argument quality. There was no main effect of either argument direction (*F*[1,249] = 1.30, *p* = .26, $\eta_{p}^{2}$ = .005) or participant’s voting intention (*F*[1,249] = .27, *p* = .60, $\eta_{p}^{2}$ = .001), indicating a comparison of the passages’ effects on endorsement of lay theories was appropriate.

#### Sociodemographic factors and lay gender theories

As expected, the Social and Biological Theory subscales were negatively correlated, *r*(281) *=* -.70, *p <* .001. Across the sample, women scored higher on the Social Theory scale (*M =* 4.11, *SD =* 1.16) than men (*M =* 3.90, *SD =* 1.47), but this difference was not significant, *t*(271) = 1.29, *p =* .20, *d =* .16. Conversely, men scored higher on the Biological Theory scale (*M =* 3.73, *SD =* 1.40) than women (*M =* 3.59, *SD =* 1.20), but again this was non-significant, *t*(271) = -.84, *p =* .40, *d =* .10.

Those with university-level education did not show significantly different scores from those of lower educational levels on the Biological Theory scale, *t*(278) = -1.37, *p =* .17, *d =* .17. However university-educated participants were significantly less positive regarding Social Theory (*M =* 3.89, *SD =* 1.31) than non-university-educated participants (*M =* 4.28, *SD =* 1.25), *t*(278) = 2.44, *p =* .02, *d =* .30.

A series of bivariate correlations were performed to establish relationships between gender theories and age, political orientation and religiosity. Higher scores on Biological Theory were correlated with greater conservatism (*r*[280] *=* .45, *p <* .001), higher religiosity (*r*[278] *=* .23, *p <* .001) and older age (*r*[275] *=* .22, *p =* .001). Conversely, higher belief in Social Theory was correlated with more liberal views (*r*[280] *=* -.48, *p <* .001), lower religiosity (*r*[278] *= -*.25, *p <* .001) and younger age (*r*[275] *=* -.29, *p <* .001).

Yes voters had significantly greater belief in Social Theory (*M =* 4.36, *SD =* 1.21) than No voters (*M =* 2.92, *SD =* .94), *t*(265) = -7.78, *p <* .001, *d =* 1.35. Yes voters reported significantly lower belief in Biological Theory (*M =* 3.35, *SD =* 1.21) than No voters (*M =* 4.82, *SD =* .95), *t*(266) = 8.03, *p <* .001, *d =* 1.33.

#### Effect of experimental manipulation

Seventeen participants failed the attention/memory check and were excluded from further analysis. Participants who responded ‘Unsure’ for their voting intention (*n =* 16) were also excluded. Despite the uneven numbers of Yes and No voters, Levene’s tests for equality of variances indicated the group variances did not significantly differ. We conducted two separate ANCOVAs to assess the effect of voting intention (Yes/No) and experimental manipulation (argument direction Yes/No) on both dependent variables (Biological and Social Theory endorsement). Gender, age, religiosity, political orientation, certainty of voting intention, and perceived strength of the argument were all potential covariates for these models. For the first step in each analysis, we tested the assumptions of homogeneity of regression slopes with each covariate. Specifically, we assessed interaction effects between each covariate and the two independent variables in each model (including the three-way interaction term with both). For both models, age and political orientation interacted with either one of the IVs. Thus, only gender, religiosity, vote certainty and argument strength were deemed appropriate covariates. The same pattern of results emerges if all six variables are included as covariates in each model.

We first investigated the effect of voting intention and argument direction on Biological Theory endorsement. There was a significant effect of the covariates argument strength (*F*[1,235] = 40.99, *p <* .001, $\eta_{p}^{2}$ = .15) and gender (*F*[1,235] = 3.96, *p =* .048, $\eta_{p}^{2}$ = .02), but not vote certainty (*F*[1,235] = 1.10, *p =* .30, $\eta_{p}^{2}$ = .005) or religiosity (*F*[1,235] = .20, *p =* .66, $\eta_{p}^{2}$ = .001). After controlling for these covariates, there was no significant main effect of argument direction, *F*(1,235) = .30, *p =* .58, $\eta_{p}^{2}$ = .001, but there remained a main effect of voting intention, *F*(1,235) = 34.73, *p <* .001, $\eta_{p}^{2}$ = .13. This main effect should be interpreted in light of a significant voting intention × argument direction interaction, *F*(1,235) = 13.45, *p <* .001, $\eta_{p}^{2}$ = .05.

**Fig 1:**
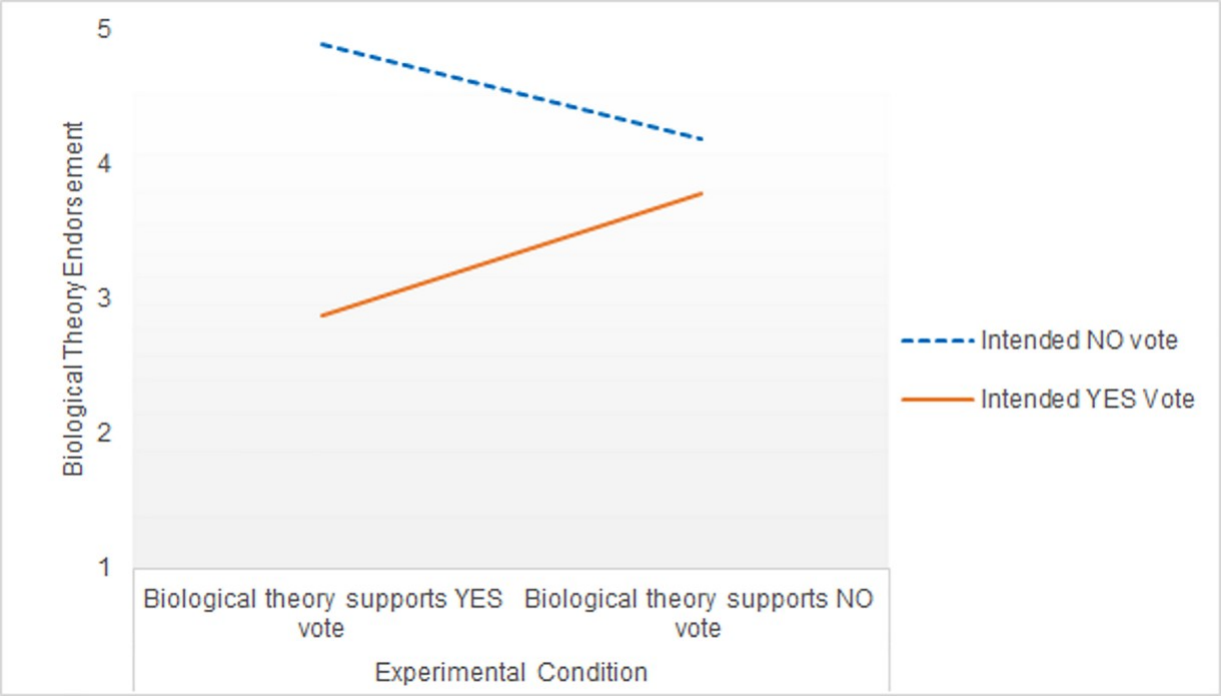
Biological Theory endorsement across voting group and experimental condition in Study 1.

Fig 1 displays the estimated marginal means underlying this interaction. It shows the pattern of effects running in the opposite direction than hypothesised. Specifically, Yes voters who read that a biological account of sex differences supported a Yes vote subsequently showed *less* endorsement of Biological Theory than Yes voters who read that biological account supported a No vote. Simple effects analysis confirmed this difference across argument conditions among Yes voters was significant, *F*(1,235) = 22.75, *p* < .001, $\eta_{p}^{2}$ = .09. Correspondingly, No voters who read that biological theories were inconsistent with their own position showed *higher* endorsement of Biological Theory than No voters who read that a biological account supported their position. However, simple effects analysis indicated this difference among No voters was non-significant, *F*(1,235) = 3.69, *p* = .06, $\eta_{p}^{2}$ = .02.

In a second ANCOVA, we investigated endorsement of Social Theory as a dependent variable. Here there was a significant effect of the covariate argument strength (*F*[1,235] = 12.23, *p =* .001, $\eta_{p}^{2}$ = .05), but not gender (*F*[1,235] = 3.19, *p =* .08, $\eta_{p}^{2}$ = .01), vote certainty (*F*[1,235] = 2.18, *p =* .14, $\eta_{p}^{2}$ = .01) or religiosity (*F*[1,235] = .18, *p =* .67, $\eta_{p}^{2}$ = .001). After controlling for these covariates, there was no significant main effect of argument direction (*F*[1,235] = 1.87, *p =* .17, $\eta_{p}^{2}$ = .01), but a significant main effect of voting intention *(F*[1,235] = 23.67, *p <* .001, $\eta_{p}^{2}$ = .09) indicated that Yes voters had higher agreement with Social Theory than No voters. The voting intention × argument direction interaction was not significant, *F*(1,235) = 2.79, *p =* .10, $\eta_{p}^{2}$ = .01.

**Fig 2:**
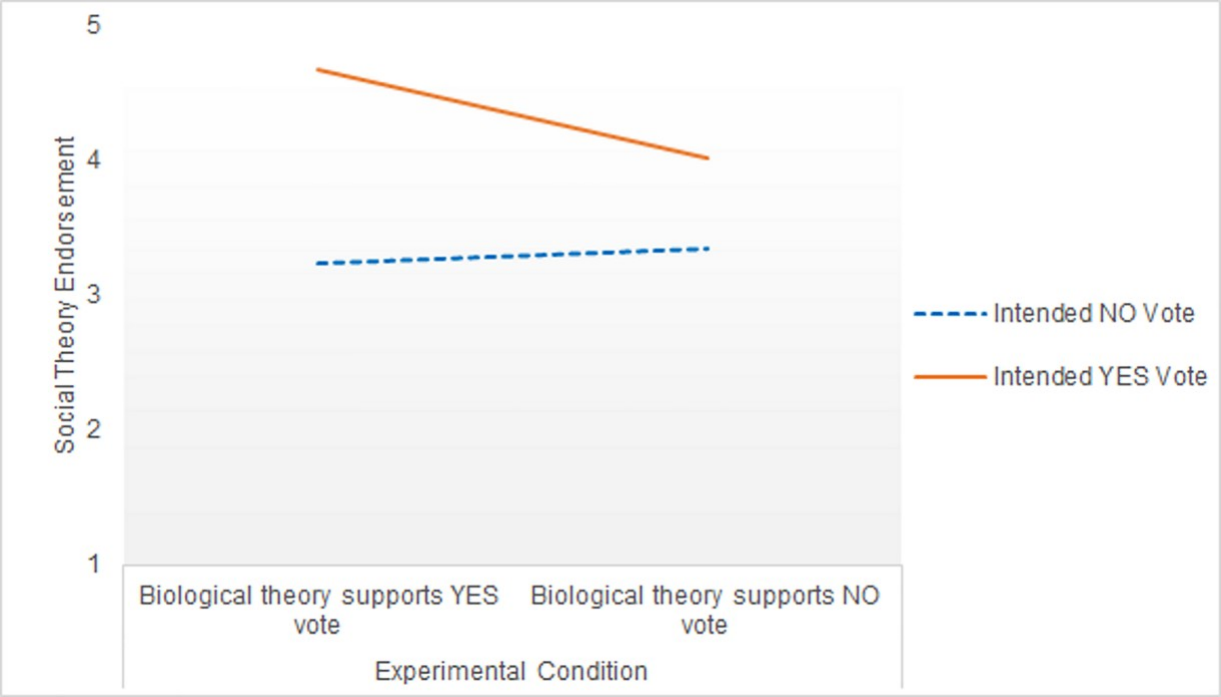
Social Theory endorsement across voting group and experimental condition in Study 1.

Although the interaction was non-significant, the pattern of expected marginal means is consistent with the results for Biological Theory (Fig 2). Yes voters who read that biological theories were consistent with a Yes vote showed higher endorsement of Social Theory than Yes voters who read that a biological account supported a No position. Simple effects analysis revealed that this difference among Yes voters was statistically significant, *F*(1,235) = 10.83, *p* = .001, $\eta_{p}^{2}$ = .04. There was no significant difference across argument conditions among No voters, *F*(1,235) = .08, *p* = .77, $\eta_{p}^{2}$ = .00.

### Interim discussion

Results ran counter to those hypothesised. Participants who read that a biological account of gender supported their own intended voting position subsequently showed lowered agreement with biological gender theories, relative to participants who were told that biological theories conflicted with their stated voting intention. This pattern held for both Yes and No voters, although stronger results were observed for Yes voters.

Given the theoretically surprising nature of these results, and their conflict with previous results showing biological gender beliefs are selectively endorsed to support ideological motivations [9,22], it is possible these findings are spurious. Study 2 therefore replicated the procedure of Study 1, but instead of reading a passage asserting the biological basis of gender, participants read a passage asserting the social origins of gender. Belief in social determinism has been proposed to operate according to similar principles as biological essentialism [77]. As in Study 1, the social account of gender was contrived to support either a Yes or No vote in the referendum.

## Study 2

Study 2 investigated the effects on lay gender theories of exposure to an experimental manipulation indicating that social theories of gender were consistent vs. inconsistent with participants’ stated voting intention.

### Method

#### Design

The structure of the questionnaire was the same as Study 1. However, instead of reading an account of biological theories of gender, participants read a 201-word passage that strongly emphasised social explanations of gender differences (S1 Appendix). As with Study 1, participants were randomly assigned to read extracts arguing that this social account of gender supported either a Yes (*N =* 107) or No (*N =* 103) vote in the referendum.

#### Participants

Recruitment strategies were the same as for Study 1. Efforts were made to advertise Studies 1 and 2 via different online communities to avoid overlap in participant pools. As anonymity concerns prevented collection of identifying characteristics or metadata, it is impossible to guarantee no duplication of participants between the studies. However, as both studies were advertised using identical wording and had a common ‘landing’ information page, they would have appeared to anyone who encountered both to be a single study; there would therefore have been little incentive to opt into both studies. A total of 241 people participated in Study 2. Participants were aged between 18–71, with a mean of 28.44 years (*SD =* 9.43). The sample was 60.9% (*n =* 109) female and 77.1% (*n =* 138) university-educated. The majority were single (68.5%, *n =* 122) and childless (79.3%, *n =* 142) at the time of research. Mean political orientation (1 = ‘extremely liberal’, 5 = ‘extremely conservative’) was 2.12 (S*D =* .98). When asked to define their religion, 37.6% (*n =* 62) identified as Christian and 60.6% (*n =* 100) atheist, agnostic or non-practicing. Mean religiosity was 1.67 (*SD =* 1.00).

#### Analysis

The analytic approach used in Study 1 was repeated. Cronbach’s α for the Biological Theory subscale was .85 and for Social Theory .83.

### Results

#### Voting intentions

Overall, 77.2% (*n* = 186) intended to vote Yes, 18.7% (*n* = 45) No and 4.15% (*n* = 10) were undecided. Most participants—96.2% (*n* = 179) of Yes voters and 81.8% (*n* = 47) of No voters—were ‘absolutely certain’ they would vote according to their stated intention.

#### Sociodemographic factors and lay gender theories

As in Study 1, the Social and Biological Theory subscales were negatively correlated, *r*(182) = -.69, *p <* .001. Women scored significantly higher on the Social Theory scale (*M =* 4.41, *SD =* 1.28) than men (*M =* 3.60, *SD =* 1.49), *t*(172) = 3.80, *p <* .001, *d =* .58. Men endorsed Biological Theory (*M =* 4.06, *SD =* 1.40) significantly more than women (*M =* 3.42, *SD =* 1.24), *t*(172) = -3.11, *p =* .002, *d =* .48.

Participants with university-level education did not show significantly different scores from those of lower educational levels on Social Theory, *t*(177) = -1.45, *p =* .15, *d =* .26. However, university-educated participants showed significantly lower belief in Biological Theory (*M =* 3.50, *SD =* 1.33) than non-university-educated participants (*M =* 4.07, *SD =* 1.26), *t*(177) = 2.45, *p =* .02, *d =* .44.

As in Study 1, agreement with Social Theory was correlated with more liberal views (*r*[178] = -.58, *p <* .001), lower religiosity (*r*[178] *= -*.23, *p =* .002) and younger age (*r*[179] *=* -.18, *p =* .02). Higher endorsement of Biological Theory was correlated with greater conservatism (*r*[178] *=* .58, *p <* .001) and higher religiosity (*r*[178] *=* .26, *p =* .001), but the correlation with age was not statistically significant (*r*[179] *=* .14, *p =* .07).

Yes voters had significantly higher endorsement of Social Theory (*M =* 4.53, *SD =* 1.20) than No voters (2.67, *SD =* 1.25), *t*(171) = -8.00, *p <* .001, *d =* 1.53. They also reported significantly lower belief in Biological Theory (*M =* 3.30, *SD =* 1.24) than No voters (*M =* 4.82, *SD =* .98), *t*(171) = 6.57, *p <* .001, *d =* 1.36.

#### Effect of experimental manipulation

Participants who failed the attention/memory check (*n* = 14) or were unsure of their voting intentions (*n* = 10) were excluded from further analysis. As in Study 1, two ANCOVAS investigated whether voting intention and argument direction affected gender theories. For each analysis, we tested the assumption of homogeneity of regression slopes with six potential covariates (gender, age, religiosity, political orientation, vote certainty, and perceived argument strength). When Social Theory endorsement was assessed as the DV, the assumption was again violated for age and political orientation, which had significant interactions with voting intention. These variables were excluded from the Social Theory ANCOVA (inclusion of these covariates did not change the pattern of results in either model). For Biological Theory endorsement, both political orientation and religiosity significantly interacted with voting intention and were deemed inappropriate covariates.

**Fig 3:**
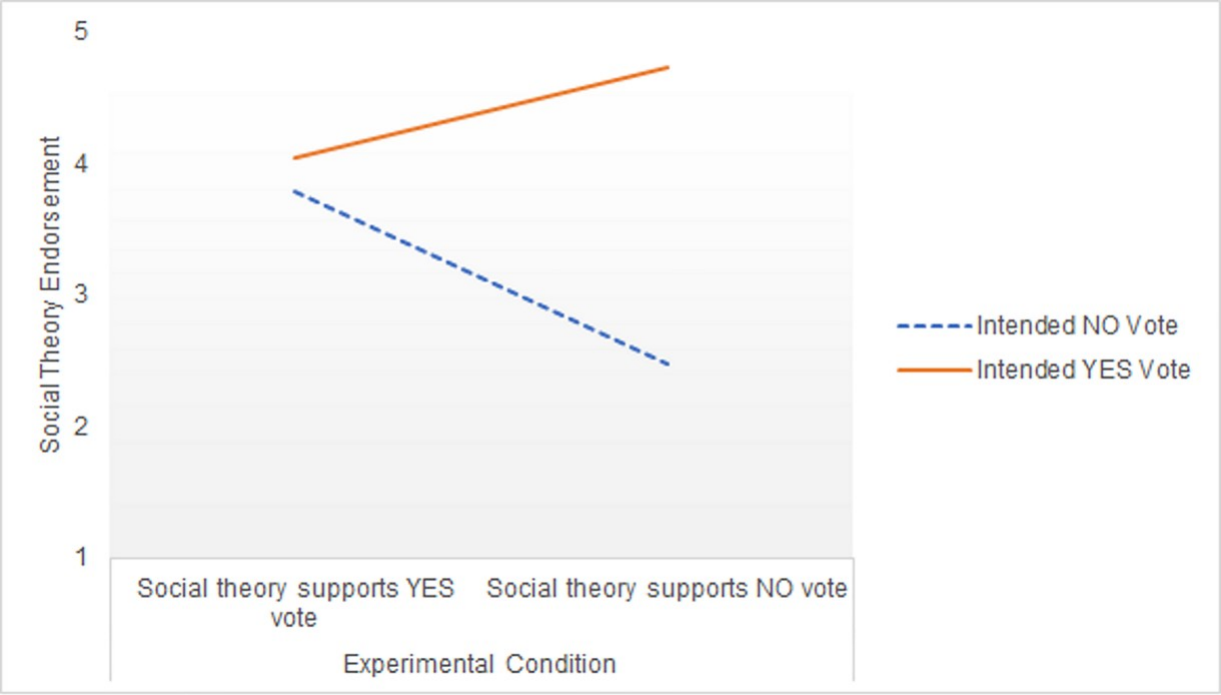
Social Theory endorsement across voting group and experimental condition in Study 2.

For the Social Theory DV, there was a significant effect of the covariates argument strength (*F*[1,142] = 20.82, *p* < .001, $\eta_{p}^{2}$ = .13) and gender (*F*[1,142] = 5.70, *p =* .02, $\eta_{p}^{2}$ = .04), but not vote certainty (*F*[1,142] = .31, *p =* .58, $\eta_{p}^{2}$ = .002) or religiosity (*F*[1,142] = .36, *p =* .55, $\eta_{p}^{2}$ = .003). After controlling for these covariates, there was no significant main effect of argument direction, *F*(1,142) = 1.46, *p =* .23, $\eta_{p}^{2}$ = .01. However, there was a significant main effect of voting intention (*F*[1,142] = 16.34, *p <* .001, $\eta_{p}^{2}$ = .10) and there was again a significant vote × argument direction interaction, *F*(1,142) = 9.68, *p =* .002, $\eta_{p}^{2}$ = .06. Fig 3 displays the estimated marginal means. Similar to Study 1’s findings, voters who read that a social account of gender supported an opposing vote subsequently showed *higher* endorsement of Social Theory compared to voters who read that a social account supported their own intended vote. Simple effects analysis revealed that these differences in endorsement of Social Theory across argument condition were significant among both No voters (*F*[1,142] = 7.31, *p* = .008, $\eta_{p}^{2}$ = .05) and Yes voters (*F*[1,142] = 4.538, *p* = .04, $\eta_{p}^{2}$ = .03).

For the Biological Theory DV, there was a significant effect of the covariate argument strength (*F*[1,143] = 4.84, *p* = .03, $\eta_{p}^{2}$ = .03), but not gender (*F*[1,143] = 2.03, *p =* .16, $\eta_{p}^{2}$ = .01), vote certainty (*F*[1,143] = .43, *p =* .52, $\eta_{p}^{2}$ = .003) or age (*F*[1,143] = 2.64, *p =* .11, $\eta_{p}^{2}$ = .02). After controlling for these covariates, there was a significant main effect of voting intention (*F*[1,143] = 15.05, *p <* .001, $\eta_{p}^{2}$ = .10), but no main effect of argument direction (*F*[1,143] = .15, *p =* .70, $\eta_{p}^{2}$ = .001). There was again a significant voting intention x argument direction interaction, *F*(1,143) = 4.17, *p =* .04, $\eta_{p}^{2}$ = .03.

**Fig 4:**
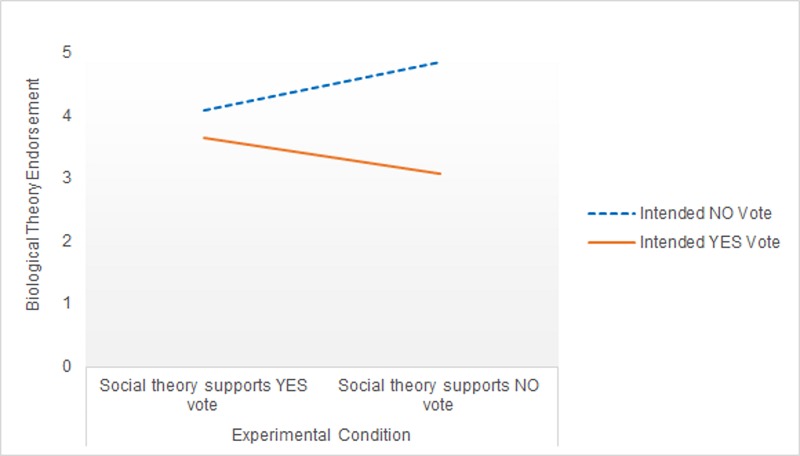
Biological Theory endorsement across voting group and experimental condition in Study 2.

The direction of the estimated marginal means shows the same pattern as previous results (Fig 4). No voters who read that a social account of gender supported a No vote reported *higher* endorsement of the Biological Theory scale than No voters who read that social account supported a Yes vote. Correspondingly, Yes voters who read that social theories were inconsistent with their own position showed lower endorsement of Biological Theory than Yes voters who read that a social account supported their position. However, simple effects analysis revealed no significant difference in Biological Theory endorsement across argument condition for Yes (*F*[1,143] = 2.87, *p* = .09, $\eta_{p}^{2}$ = .02) or No voters (*F*[1,143] = 2.44, *p* = .12, $\eta_{p}^{2}$ = .02).

### Interim discussion

Study 2 shows the same pattern of results as Study 1. For both Yes and No voters, reading that a social account of gender was consistent with one’s own voting intention reduced support for social theories of gender, relative to participants who read that the social theory was inconsistent with their voting intention.

## Discussion

The Irish abortion referendum of 2018 was a unique cultural, political and historical event. The current research took advantage of this opportunity to explore the relationship between abortion attitudes and lay theories of gender. Across both studies, those who wished to liberalise Ireland’s abortion regime endorsed more social and less biological theories of gender than those who wished to maintain the abortion ban. The co-incidence of certain gender theories and abortion attitudes has not previously been demonstrated in the published literature, but is unsurprising given prior evidence that biological essentialism is linked with more conservative worldviews [6,8,10]. The two studies reported in this paper sought to enlighten the causal directionality of this relationship by testing whether exposure to gender theories, which were presented as attitude-consistent vs. attitude-inconsistent, affected people’s causal gender beliefs. Based on previous research showing causal gender attributions are mutable to identity and ideological motivations [9,22], we hypothesised that participants would more strongly endorse gender theories that were presented as supporting their pre-existing voting attention. However, the opposite effect emerged: participants subsequently showed higher endorsement of gender theories portrayed as *conflicting* with their voting intention. This pattern of effects was consistent across both studies (that exposed people to social and biological accounts of gender), Yes and No voters, and both dependent variables (endorsement of social and biological theories).

These results diverge from previous research showing motivated endorsement of essentialist theories [9,21,22,78], as well as the vast body of social cognitive research showing biased assimilation of attitude-consistent information [79]. It is, of course, possible that the findings are spurious. However, a number of features of the current research preclude its wholesale dismissal, including its reasonably large sample; the elicitation of data on attitudes and behavioural intentions pertaining to a meaningful, topical and consequential issue; and the consistency of results across studies, dependent variables and experimental conditions.

The time-specific nature of the research, which had a three-week data collection window before the referendum occurred, unfortunately precluded the initiation of further studies to investigate the social psychological mechanisms underlying the unexpected findings. Interpretation of the results therefore remains speculative. One possibility is that the results reflect a reactance or ‘boomerang’ effect resulting from a persuasion attempt perceived to be too heavy-handed [80]. The study took place at a time when national media was saturated with referendum coverage, as both campaigns clamoured to broadcast their message to the electorate. In this context, participants may have perceived the study as yet another attempt to manipulate them, and expressed their frustration by shifting in the opposite direction than expected of them. The referendum campaign was also marked by frequent appeals for ‘balance’, exemplified by an obligation on radio and television broadcasters to ensure programming fairly represented the interests of both sides of the debate. Given these norms, it is possible participants in this study valued exposure to arguments with which they did not personally agree [81]. These issues highlight the methodological challenges in designing experimental research pertaining to topical, real-world socio-political events: gains in ecological validity may come at the cost of the data’s liability to unpredictable and uncontrollable socio-emotional motives.

Another potential explanation relates to the high levels of certainty participants reported regarding their voting intention. Despite previous evidence that abortion attitudes typically show high levels of ambiguity and contextuality [46,82,83], the referendum presented Irish citizens with a binary choice. Over 90% of Yes-voting participants rated themselves ‘absolutely certain’ they would vote this way. This may have attenuated any motivation to engage in biased cognition, as participants did not need additional justification for their already secure position. The normative nature of their voting intention within their social networks, particularly for Yes voters, may also have reduced motivation to bolster their position. This close to the referendum, participants had likely already been exposed to a necessary and sufficient quantity of arguments to justify their position: an additional argument based on gender theories may have been extraneous. This would explain a null effect in Studies 1 and 2, but does not quite account for the backlash effect that was observed. One possibility is that participants in the attitude-consistent conditions compared the gender theory arguments to the other arguments supporting their position they had encountered in real life, and judged the gender theory arguments weak in comparison. This evaluation of the quality of the gender theory arguments for/against abortion may have generalised to the premise of the gender theory itself, independent of its relation to the abortion issue. Due to self-selection into social circles and media outlets where one’s own attitude is shared, participants in the attitude-inconsistent conditions may not have encountered as many arguments for that position. Without a frame of reference to evaluate the relative quality of attitude-inconsistent arguments, the inherent weaknesses of the gender theory argument may have been less apparent.

If participants’ high attitudinal certainty meant the context was not conducive to motivated reasoning, the results may be better interpreted on a more basic cognitive or information processing level. Participants were initially shown specific gender theories, followed by the introduction of pro-life/pro-choice arguments. In each study, the findings demonstrated higher endorsement of the presented gender theories among participants who were subsequently exposed to an argument that conflicted with their voting preference. This may reflect an ideological priming effect among these groups [84]. Such priming effects should be interpreted with regard to cognitive and motivational processing resources [85,86]. Numerous psychological models predict that, in general terms (and in the absence of motivated reasoning), fluent information processing leads to subtle positive changes in core affect, which in turn facilitate the activation of intuitive [87,88] or chronically accessible [89,90] attitudes. On the other hand, incongruent information and negative affect increase cognitive processing resources [91,92] and reduce reliance on ‘default’ or chronically accessible attitudes. In this research, participants’ existing lay theories of gender could be considered as chronically accessible ideological attitudes [90,93]. Thus, when the arguments presented were congruent with participants’ own voting intention, information was processed fluently and the expected discrepancy in gender theories between Yes- and No-voters was evident. However, the presentation of arguments incongruent with participants’ voting intentions may have elicited negative affect, which created a priming effect by facilitating the inclusion of temporarily accessible information (the gender theories just encountered) in participants’ attitudes. In other words, the initial exposure to the biological (or social) accounts primed stronger endorsement of biological (or social) theories among voters subsequently exposed to arguments that conflicted with their voting preferences.

Whatever the potential mechanism for the results observed, they do not support the hypothesis that participants’ endorsement of gender theories was motivated by desire to bolster existing socio-political attitudes regarding abortion. However, neither do they indicate that gender theories were entirely independent of abortion attitudes: the experimental manipulations did significantly affect endorsement of gender theories, albeit in the opposite direction than expected. This provides further evidence for the principle that lay gender theories are mutable, although the mechanism driving their adaptation in this research remains unclear.

### Strengths and limitations

One weakness of this research relates to the quality of the experimental manipulations, i.e. the gender theory-based pro-life and pro-choice arguments to which participants were exposed. The presentation of fabricated biological/social accounts of gender differences is common practice in experimental research seeking to manipulate levels of essentialism [6,9,11,19]. Development of the passages was informed by inspection of real-world media content and academic literature on abortion discourse in Ireland. For example, the passages incorporated the central role of ‘choice’ in abortion discourse [73] and Irish anti-abortion advocates’ focus on the social circumstances seen to promote abortion, such as inadequate childcare provision and economic deprivation [76]. The statistical analyses did control for the perceived strength of the arguments. Nevertheless, it is possible that the specific arguments developed for this research were not seen by participants as credible, weakening their power as experimental manipulations.

The research was also subject to sampling limitations. Young, female, educated and non-religious people were over-represented in both studies. Yes-voters were also over-represented relative to the final referendum result (66.4% Yes). Recruitment took place online, which restricted the sample to those active on the platforms where the research was advertised. Recruitment specifically targeted interest-groups already engaged in the referendum debate (e.g. by placing adverts on relevant discussion threads). This bias was intentional, as the experimental design required people with existing commitment to a certain voting intention. It also increased response rates by targeting people interested in the topic. However, these populations may have had distinctive demographic or psychological profiles that affected the results gleaned.

Neither study included a control condition that measured Yes- and No-voters’ baseline gender theory beliefs, independent of any experimental manipulation. Without such data, it is difficult to judge whether the interaction effects obtained were primarily driven by changes in the attitude-consistent condition, attitude-inconsistent condition, or both. Neither was voting intention re-assessed to establish whether intentions were affected by the experimental manipulation (although participants’ high expressed certainty in their intended vote probably made this unlikely). Given ethical requirements to immediately debrief participants (so as not to influence their actual voting behaviour), it was not possible to collect follow-up data to establish whether the experimental effects persisted. Ethical concerns regarding not interfering with citizens’ voting behaviour in the approaching referendum also prevented any investigation of the reverse direction of the gender theories–abortion attitudes relationship, namely whether manipulation of causal gender attributions would shift abortion attitudes. This may be an appropriate focus of investigation for future research in less immediately consequential socio-political environments.

Research in other contexts would also be welcome to clarify the influence of the unique circumstances of the Irish referendum on the results obtained. It is worth noting that public debate preceding the Irish referendum largely focused on cases of abortion due to maternal risk or fatal foetal abnormality, i.e. ‘traumatic’ rather than ‘elective’ abortion. Previous research suggests gender-related beliefs are stronger predictors of attitudes to elective rather than traumatic abortion [94]. If participants’ voting intentions were predominantly driven by attitudes to traumatic abortion, the relevance of lay gender theories may have been minimised. Additionally, although religious observance has declined dramatically in Ireland since the 1990s, the traditionally strong Catholic influence on Irish culture may mean beliefs about the status and rights of the foetus overshadow beliefs about gender in determining abortion attitudes [47–49]. Lay theories of human categories beyond gender may prove a fruitful avenue for investigation for future research.

The studies’ limitations notwithstanding, the research contributes the first quantitative analysis of the relationship between lay theories of gender and abortion attitudes. It expands the body of research that has approached abortion from a social psychological lens and broadens the international scope of the literature on abortion attitudes. Its setting within a meaningful socio-political event heightens its relevance and validity, and it is one of the first studies to relate biological essentialism to behavioural intentions regarding a topical socio-political issue, rather than generic attitudinal measures. The demonstrated mutability of gender theories is consistent with the argument that causal attributions primarily function to communicate ideological and political meanings, rather than constitute social attitudes [75]. However, the patterns of results in both studies throw doubt on the proposition that the endorsement of particular gender theories is simply determined by their alignment with pre-existing attitudinal commitments. Further cross-sectional and experimental research is required to understand the basis for the coherence between particular theories of gender and attitudinal positions regarding abortion.

## Supporting information

S1 Appendix Study materials. (PDF)

S1 Dataset Study 1 dataset. (SAV)

S2 Dataset Study 2 dataset. (SAV)
