# Supplementary material for: Exploring the relationship between lay theories of gender and attitudes to abortion in the context of a national referendum on abortion policy
Source: PLoS One. 2019 Jun 13;14(6):e0218333. doi: 10.1371/journal.pone.0218333 (PMC6564017; doi:10.1371/journal.pone.0218333)
Supplement: S1 Appendix — (PDF) [file pone.0218333.s001.pdf]

S1 Appendix: Study Materials

Experimental passages

Study 1: Biological theory passage

Fundamentally, gender is a biological phenomenon. Gender is best defined as a binary category, whereby individuals' genetic profiles establish them as either male or female. Scientific research shows that gender differences in behaviour and emotions are large in size and are best explained by biological factors, such as hormonal processes. In human cultures across the world, it is universally and consistently women who take the primary role in caring for children.

YES (pro-choice) conclusion: This has distinct implications for the upcoming referendum to expand access to abortion by repealing the Eighth Amendment of the Irish Constitution. Due to the biological role women play in human reproduction, the burden of an unplanned or high-risk pregnancy falls disproportionately on them. They should therefore have the power to deal with that pregnancy in a way that is right for them as women. The experience of living in a female body gives women a unique, intuitive sense of what is right for their and their families' welfare. Only women can judge whether they are capable of giving a child the life it deserves. Society must respect women's natural instincts about whether, when and how they wish to have a child. We should vote YES in the upcoming referendum.

NO (pro-life) conclusion: This has distinct implications for the upcoming referendum to expand access to abortion by repealing the Eighth Amendment of the Irish Constitution. Due to the biological role women play in human reproduction, they are endowed with an innate drive to nurture and nourish children. Women who seek abortion do so out of desperation due to poverty, mental health problems, dysfunctional family contexts and worries about childcare.

The best way to protect women's welfare is to tackle these root causes, and implement policies that support women in realising their natural maternal instincts. Proposals to widen access to abortion disregard the innate strength women find when they become mothers. Society must celebrate motherhood and respect the unique place it occupies in the natural order. We should vote NO in the upcoming referendum.

Study 2: Social theory passage

Fundamentally, gender is a social phenomenon. Gender is best defined as a continuum, whereby individuals' upbringings afford them different levels of 'masculine' and 'feminine' traits. Scientific research shows that gender differences in behaviour and emotions are small in size and are best explained by social factors, such as cultural expectations. In human cultures around the world, there are large variations in the roles men and women take in caring for children.

YES (pro-choice) conclusion: This has distinct implications for the upcoming referendum to expand access to abortion by repealing the Eighth Amendment of the Irish Constitution. The cultural variability of gender roles highlights the importance of the social environment in determining people's lives. Due to the position women currently hold in Irish society, the burden of an unplanned or high-risk pregnancy falls disproportionately on them. This unfairly disadvantages their ability to attain a rewarding career, financial stability and fulfilling personal life. The best way to promote women's welfare is to afford them the power to deal with a pregnancy in a way that is right for them as individuals. Society should respect individuals' personal decisions about whether, when and how they wish to have a child. We must vote YES in the upcoming referendum.

***NO* (pro-life) conclusion**: This has distinct implications for the upcoming referendum to expand access to abortion by repealing the Eighth Amendment of the Irish Constitution. The cultural variability of gender roles highlights the importance of the social environment in determining people's lives. Due to the position women currently hold in Irish society, the burden of an unplanned or high-risk pregnancy falls disproportionately on them. Women who seek abortion do so out of desperation due to poverty, mental health problems, dysfunctional family contexts and worries about childcare. The best way to promote women's welfare is to tackle these root social causes; simply ending pregnancies will not solve and may even exacerbate these issues. Society should not be distracted from the real social causes of human distress. We must vote NO in the upcoming referendum.

Questionnaire

On the 25th of May, there will be a referendum to decide the legal basis for abortion in Ireland. Irish citizens will vote on whether to amend the Constitution by removing the Eighth Amendment and inserting legal provision for the regulation of termination of pregnancies.

What way are you likely to vote in this referendum?

- ○ YES (remove the Eighth Amendment restrictions on abortion)
- ○ NO (retain the Eighth Amendment restrictions on abortion)
- ○ Don't know/undecided

On a scale of 1-4, how certain are you that you will vote this way?

- ○ 1 (Not certain at all)
- ○ 2
- ○ 3
- ○ 4 (Absolutely certain)

<EXPERIMENTAL PASSAGE (randomly assigned)>

How strong do you find the above argument? Please evaluate the quality of the argument on a scale from 'Extremely weak' to 'Extremely strong'. Insofar as possible, please try to evaluate the argument on its own merits, leaving aside your personal views.

- ○ Extremely weak
- ○ Somewhat weak
- ○ Neither weak nor strong
- ○ Somewhat strong
- ○ Extremely strong

- -

The text presented on the previous page was arguing in favour of:

- ○ a YES vote in the upcoming referendum (i.e. to remove the restrictions on abortion imposed by the Eighth Amendment)
- ○ a NO vote in the upcoming referendum (i.e. to retain the restrictions on abortion imposed by the Eighth Amendment)
- ○ not sure/don't remember

- -

Below is a series of statements about gender. Please rate your level of agreement with each statement using the scales provided.

	Strongly disagree	Disagree	Somewhat disagree	Neither agree nor disagree	Somewhat agree	Agree	Strongly agree
To a large extent, a person's gender biologically determines his or her abilities and traits	○	○	○	○	○	○	○
It is hard if not impossible to change the innate dispositions of a person's gender	○	○	○	○	○	○	○
When men and women differ in some way, it is likely that the difference is due to biological factors	○	○	○	○	○	○	○
The innate properties of a person's gender determine what the person is like	○	○	○	○	○	○	○
The properties of gender are constructed totally for economic, political, and social reasons	○	○	○	○	○	○	○

If social situations change, the characteristics we attribute to gender categories will change as well	○	○	○	○	○	○	○
Gender is not set in stone and can be changed	○	○	○	○	○	○	○
Gender is a result of “nurture” more than “nature”	○	○	○	○	○	○	○
A person's gender has more to do with a person's social environment than with an individual's disposition	○	○	○	○	○	○	○
Gender is more directly linked to biology than to the way a person is socialised	○	○	○	○	○	○	○
People's displays of gender behaviours are based more on biological factors than on the social climate	○	○	○	○	○	○	○

What is your gender?

- ○ Woman
- ○ Man
- ○ Other/Prefer not to say

What is your year of birth?

____

What is your nationality?

____

What is your marital status?

- ○ Single
- ○ Married
- ○ Living with partner
- ○ In a committed relationship
- ○ Divorced/separated
- ○ Widowed

Do you have children?

- ○ Yes
- ○ No

What is your religion (if any)?

____

How important is religion to you personally?

- ◯ Extremely important
- ◯ Very important
- ◯ Moderately important
- ◯ Slightly important
- ◯ Not at all important

People's views on social issues can be characterised as liberal (left-wing) or conservative (right-wing). Where would you generally place yourself on this scale?

- ◯ Extremely liberal
- ◯ Somewhat liberal
- ◯ Neither liberal nor conservative
- ◯ Somewhat conservative
- ◯ Extremely conservative

What is the highest level of education you have completed?

- ◯ Primary school
- ◯ Lower secondary (e.g. Junior Certificate)
- ◯ Higher secondary (e.g. Leaving Certificate)
- ◯ Technical or vocational qualification
- ◯ University degree (undergraduate)
- ◯ Postgraduate degree
